# The Relationship between the Occupational Exposure of Trichloroethylene and Kidney Cancer

**DOI:** 10.1186/2052-4374-26-12

**Published:** 2014-06-03

**Authors:** Inah Kim, Jaehyeok Ha, June-Hee Lee, Kye-mook Yoo, Jaehoon Rho

**Affiliations:** 1 Department of occupational health, Yonsei University Graduate School of Public Health, Seoul, Korea; 2 Institutes for Occupational Health, Yonsei University College of Medicine, Seoul, Korea; 3 Department of occupational health, Seoul National University Graduate School of Public Health, Seoul, Korea; 4 Occupational Safety and Health Research Institute, Korea Occupational Safety and Health Agency, Ulsan, Korea; 5 Department of Preventive Medicine, Yonsei University College of Medicine, Seoul, Korea

**Keywords:** Trichloroethylene, Kidney cancer, Korea, Work-related, Occupation, Exposure

## Abstract

Trichloroethylene (TCE) has been widely used as a degreasing agent in many manufacturing industries. Recently, the International Agency for Research on Cancer presented “sufficient evidence” for the causal relationship between TCE and kidney cancer. The aim of this study was to review the epidemiologic evidences regarding the relationship between TCE exposure and kidney cancer in Korean work environments. The results from the cohort studies were inconsistent, but according to the meta-analysis and case–control studies, an increased risk for kidney cancer was present in the exposure group and the dose–response relationship could be identified using various measures of exposure. In Korea, TCE is a commonly used chemical for cleaning or degreasing processes by various manufacturers; average exposure levels of TCE vary widely. When occupational physicians evaluate work-relatedness kidney cancers, they must consider past exposure levels, which could be very high (>100 ppm in some cases) and associated with jobs, such as plating, cleaning, or degreasing. The exposure levels at a manual job could be higher than an automated job. The peak level of TCE could also be considered an important exposure-related variable due to the possibility of carcinogenesis associated with high TCE doses. This review could be a comprehensive reference for assessing work-related TCE exposure and kidney cancer in Korea.

## Introduction

Trichloroethylene (TCE) has been widely used as a degreasing agent in many manufacturing industries (i.e., metal processing or electronic device production) due to its lipid solubility, volatility, no flammability, and economic efficiency [1]. TCE is a well-known carcinogen according to animal studies. The reactive metabolites of the glutathione pathway and the oxidation process of TCE could have carcinogenic effects in kidney and liver or lung, respectively [2].

Recently, the International Agency for Research on Cancer (IARC) categorized TCE as a Group 1 carcinogen and stated that there is “sufficient evidence” for the causal relationship between TCE and kidney cancer [3]. According to increasing epidemiologic evidences, the first work-related case of kidney cancer in Korea was reported in 2013 [4]. TCE is still widely used as degreasing or cleansing agents and high exposure levels of TCE could be prevalent in Korea. Moreover, the prevalence or incidence of target cancers associated with TCE (i.e., kidney cancer) has rapidly increased in the Korean population [5].

The aim of this study was to review the epidemiologic evidences regarding the relationship between TCE exposure and kidney cancer in the working population in Korea.

## Review

### Incidence and risk factors of kidney cancer

According to the national cancer statistics, the age-standardized incidence rate of kidney cancer per 100,000 persons increased from 3.1 in 1999 to 3.5, 4.9, 5.4, 7.3, and 8.0 in 2002, 2005, 2006, 2010, and 2011, respectively. Kidney cancer had the third most rapid increase (6.2%), following thyroid (23.7%) and prostate (13.5%) cancers; but the incidence of esophageal, stomach, laryngeal, and cervical cancers decreased from 1999 to 2011. The annual percentage change over time, expressed as (exp(b) - 1) × 100, where b was the estimated slope of a linear regression from a logarithmic scaled age-standardized incidence rate and calendar year, was statistically significant in both genders. In 2011, the crude incidence rate of kidney cancer per 100,000 men was 10.9 (2,722 cases), which was the 9^th^ most common cancer among Korean men, following cancers of the stomach, colon or rectum, lung, liver, prostate, thyroid, bladder, and pancreas (Table 1). The incidence of kidney cancer increased with age, but the incidence was found to be the highest for people ≥70 years, which was 44.5/100,000 for men and 16.5/100,000 for women (Table 2) [5]. The subtypes of renal cell cancer (RCC) were divided into the clear cell type, papillary type, chromophobe type, and collecting duct type. Currently, there is limited evidence to indicate that occupational risk factors increase RCCs of a specific subtype.

**Table 1:** Crude incidence rate (CR) and age-standardized incidence rate (ASR) of kidney cancer per 100,000 persons from 1999 to 2011 in Korea

	**Total**		**Men**		**Women**	
	**CR**	**ASR**	**CR**	**ASR**	**CR**	**ASR**
1999	3.0	3.1	4.1	4.7	1.9	1.7
2000	3.0	3.0	4.1	4.6	1.9	1.8
2001	3.5	3.4	4.7	5.1	2.2	2.0
2002	3.6	3.5	5.0	5.2	2.3	2.1
2003	3.9	3.6	5.3	5.4	2.5	2.2
2004	4.3	3.9	5.8	5.7	2.8	2.4
2005	4.9	4.2	6.6	6.3	3.1	2.5
2006	5.4	4.6	7.4	6.8	3.5	2.7
2007	6.0	5.0	8.3	7.4	3.7	2.9
2008	6.6	5.2	9.0	7.8	4.1	3.1
2009	7.0	5.5	9.4	7.9	4.5	3.3
2010	7.3	5.5	10.1	8.3	4.3	3.1
2011	8.0	6.2	10.9	8.6	5.1	3.6
APC*		6.2 (p<0.05)		5.9 (p<0.05)		6.3 (p<0.05)

*APC; Annual Percentage Change, expressed as (exp(b)-1)×100, where b is the estimated slope of a linear regression from a logarithmic scaled age-standardized rates and calendar years.

**Table 2:** Trends in kidney cancer incidence rates in Korea (per 100,000 persons)

	**Men**							**Women**						
**Age (years)**	**2002**	**2003**	**2004**	**2005**	**2009**	**2010**	**2011**	**2002**	**2003**	**2004**	**2005**	**2009**	**2010**	**2011**
0–9	0.6	0.6	0.7	0.6	0.8	0.9	0.8	0.8	0.5	0.5	0.6	0.6	0.7	0.7
10–19	0.0	0.1	0.3	0.1	0.1	0.1	0.1	0.0	0.1	0.0	0.1	0.1	0.1	0.2
20–29	0.4	0.4	0.4	0.6	0.8	0.7	0.7	0.4	0.3	0.3	0.3	0.6	0.3	0.6
30–39	1.9	2.0	2.1	2.2	3.8	3.9	4.3	0.9	1.2	1.1	1.0	1.3	1.7	2.1
40–49	5.4	6.7	6.5	7.1	9.4	9.8	10.3	1.9	3.0	3.0	2.5	4.1	3.7	4.0
50–59	13.7	13.0	12.4	15.0	18.4	20.5	21.1	5.3	4.3	6.3	5.6	8.1	7.7	8.5
60–69	22.7	23.2	24.7	24.5	31.8	33.1	33.6	8.1	8.9	8.1	10.1	12.8	12.3	13.5
≥70	26.2	25.4	32.4	36.5	42.2	41.5	44.5	9.6	9.5	9.8	13.0	15.3	13.6	16.5

The risk factors for RCC include smoking, obesity, a past history of renal stones [6], and the presence of a genetic mutation known as Von Hippel-Lindau syndrome, which is present in 1/3~1/2 patients [7]. Various metals (i.e., arsenics, cadmium, lead, or uranium), poly-aromatic hydrocarbons, solvents (i.e., chlorinated hydrocarbons), and asbestos have been considered as occupational risk factors for RCC in several studies [8]. According to the IARC, TCE has sufficient evidence while arsenic and inorganic arsenic compounds, cadmium and its compounds, and printing processes have limited evidences for kidney cancer in humans [8].

### Occupational exposure of trichloroethylene in Korea

#### Work environment survey in manufacturers in 2004

According to work environment survey for manufacturers, the annual amount of TCE usage was 7849 tons; 1982.4 tons by manufacturers of motor vehicles, trailers and semitrailers, 1085.9 tons by manufacturers of fabricated metal products, except machinery and furniture, 1056.4 tons by manufacturers of electric components, computer, radio, television, and communication equipment and apparatuses, and 483.8 tons by manufacturers of machinery and equipment. TCE was mostly used as a cleaning agent. About 5,949 workers in 1,540 companies could have been exposed to TCE during production processes in 2004 [9].

#### A survey on the status of using trichloroethylene

After 2 employees had died from Steven Johns syndrome related to TCE exposure in 2006, a survey on the distribution and usage of TCE was conducted using a database to work environment monitoring of the representative 103 companies. TCE was mostly used by manufacturers of motor vehicle and engine parts and accessories (23,920 L). The personal ambient exposure levels in this survey ranged from non-detectable (ND) to 49.87 ppm. The range of ambient exposure level of TCE was ND~49.87 ppm in manufacturers of motor vehicle and engine parts and accessories, 0.08~41.55 ppm in manufacturers of electric components, computer, radio, television, and communication equipment and apparatuses, and ND~30.80 ppm in manufacturers of fabricated metal products, except machinery and furniture (Table 3). Workers engaging in manual tasks or semi-automated processes were more frequently exposed to TCE than those who worked with automated processes (87% vs. 13%) [10].

**Table 3:** Exposure status according to trichloroethylene use in 2006 in Korea

**Type of industry**	**No. of factories**	**No. of workers**	**Amount used (L/month)**	**Concentration range (ppm)**
Total	103	390	87,320	-
Chemical	3	19	4,116	0.97~13.26
Plastics & rubber products	1	35	6,830	ND~42.63
Primary metal	6	18	6,787	2.91~37.35
Fabricated metal products	24	59	11,990	ND~30.80
Machinery	18	59	3,950	ND~48.48
Computer & electronic products	9	62	19,596	0.08~41.55
Electrical equipment, appliance	10	56	4,771	ND~21.29
Transportation equipment	18	30	23,920	ND~49.87
Others	14	52	5,360	ND~39.51

ND; Non-detectable, concentration level was lower than the detection limit.

(Source: Cho et al. 2007) [10].

#### Work environment monitoring

The analysis of ambient exposure levels (time weighted average (TWA) from 8 hours) of TCE from the work environment monitoring (WEM) in the manufacturing industries conducted by private occupational health organizations during 2002–2010 is shown in Table 4. A total of 33,652 samples were analyzed and we re-classified the task categories to 22 from 537. The range of the geometric mean (GM) was 0.00015~0.25311 ppm. The exposure level of TCE was highest during plating (0.25311 ppm), followed by cleaning (0.16013 ppm) and degreasing (0.04185 ppm). The highest exposure level of TCE by personal sampler was 598 ppm for cleaning, followed by 237 ppm for assembly, 154 ppm for coating, 152 ppm for degreasing, and 148 ppm for painting. The median exposure levels of TCE were 3.18 ppm, 2.33 ppm, and 2.01 ppm for plating, cleaning, and degreasing, respectively. As a result, cleaning, degreasing, and plating could be high-risk jobs for TCE exposure.

**Table 4:** Exposure levels of trichloroethylene by Korean manufacturing industry jobs according to the regular work environment measurement (2002~2010)

**Job**	**No. of samples**	**Arithmetic mean (ppm)**	**Median (ppm)**	**Geometric mean (ppm)**	**Maximum (ppm)**
Cleaning	8,374	8.953	2.33495	0.16013	598
Degreasing	421	7.780	2.01080	0.04185	152
Assembly	1,146	2.850	0.08975	0.00101	237
Adhesion	700	1.044	0.16850	0.00172	29
Coating	671	3.250	0.48790	0.01499	154
Painting	3,713	1.326	0.00000	0.00015	148
Processing	1,046	3.955	0.41855	0.01159	108
Inspection	890	2.704	0.59650	0.01061	94
Printing	1,519	1.948	0.17800	0.00518	65
Soldering	802	2.053	0.42860	0.00881	50
Plating	399	8.508	3.17970	0.25311	63
Molding	507	4.545	0.71700	0.01194	60
Laboratory	317	0.692	0.00000	0.00006	48
Impregnation	199	7.697	0.18200	0.00839	185
Mixing	511	1.807	0.09700	0.00111	54
Heat treatment	201	4.982	1.00000	0.01987	64
Plugging	34	0.565	0.08136	0.00063	8
Infusion	267	5.846	0.40630	0.01467	270
Reaction	138	0.461	0.00000	0.00005	6
Packing	273	2.128	0.27600	0.00597	45
Cast	124	2.601	0.04165	0.00060	43
Others	11,400	9.318	0.27935	0.00540	1,471
Total	33,652	6.497	0.40790	0.00840	–

#### Reliability of work environment monitoring

Considering the limitation of the reliability or validity of WEM, we analyzed reports from 16 enterprises, which were assessed for the reliability of WEM by the Korea Agency for Occupational Safety and Health for cleaning jobs, which were the most common jobs in 2006. The highest level of TCE exposure was 116.62 ppm in manufacturers of non-metallic mineral products (i.e., press and cleaning), followed by manual cleaning for optic device production (114.41 ppm), degreasing for rubber goods production (88.84 ppm), and cleaning for transport machineries and equipment production (49.86 ppm; Table 5).

**Table 5:** Exposure levels of cleaning or degreasing jobs using assessment of reliability of work environment monitoring in 2006 in Korea

**Industry in manufacturer**	**Job1**	**Job2**	**Method**	**Range of concentration (ppm)**
Electronic components (LCD panel frame)	Press	Processing & cleaning	Solid sampler. NIOSH method 1022	8.258~11.995
Power electric equipment (painting transformers)	Storing component & cleaning	Printing & cleaning	Solid sampler. NIOSH method 1022	Painting and cleaning: 0.343~9.742 masking: 1.099~16.432, touch up: 0.149~2.462
Measuring, optic and precision instrument	Press & cleaning	Grinding & cleaning	Solid sampler. diffusive sample	16.99~114.41 (including manual work)
Television and communication equipment	Press molding & processing	Assembly & cleaning	Solid sampler. NIOSH method 1022	85.44 in manual (closed 1 years ago), 2.16~3.75 in automatic
Optic equipment and lens	Cutting	Cleaning	Solid sampler. NIOSH method 1022	52.90 (in indoor)
Parts and accessories for motor vehicle	Assembly & dipping	Bonding & cleaning	Sorbent tube	11.57~18.52
Electronic components	Press	Cleaning	Sorbent tube	31.72~49.86
Textile	Degummed and twist thread	Decontamination	Sorbent tube	0.14~1.38
Plating	1^st^ cleaning	2nd cleaning	Sorbent tube	1st cleaning: 28.59, 3.55
				2nd cleaning: 39.28, 1.81
Metal tooling	Press	Cleaning	Solid sampler. NIOSH method 1022	8.75~9.22
Parts and accessories for motor vehicle	Mixing & surface treatment	Degreasing	KOSHA CODE-A-1-2004 (Method No. 016)	2.56
Other electric equipment	Wring & dipping	Assembly & impregnation	KOSHA CODE-A-1-2004 (Method No. 016)	0.03~0.05
Rubber goods production	Preparation	Degreasing	KOSHA CODE-A-1-2004 (Method No. 016)	15.49~88.84
Other metal product	Press and spot welding	Cleaning	KOSHA CODE-A-1-2004 (Method No. 016)	7.21~11.7
Transport machineries and equipment	Press and spot welding	Cleaning	KOSHA CODE-A-1-2004 (Method No. 016)	14.90~49.83
Non-metallic mineral product	Melting & extrusion	Press & cleaning	KOSHA CODE-A-1-2004 (Method No. 016)	41.32~116.62

#### Peer-reviewed and published papers in Korea

According to peer-reviewed and published papers in Korea, the exposure level of TCE in the period from 1970s’ to 1980s’ was higher than that in recent periods. Paik et al. reported that the exposure levels of TCE in 1970, were 110 ppm in the corners of the cleaning room, 124 ppm in regions where cleaning is initiated, and 221~301 ppm in regions where cleaning is terminated [11]. Kim et al. also reported that the exposure levels of TCE during the plating process in 1989: 19.8~50.3 ppm in the cleaning bath and 130.8~456.2 ppm in the drying bath [12]. The personal ambient exposure level of TCE for manual cleaning workers was 83.5 ppm and the regional ambient exposure level of TCE for manual polishing workers was 35.5 in 1989. At that time, the percentage of enterprises with ambient exposure levels higher than the occupational exposure limit set by the government (i.e., 75 ppm) was 54.2% in 1989 [13]. The GM of the 8 hours’ TWA for degreasing workers was 26 ppm and the range of the exposure level was 1.4~123 ppm. The GM of the 8 hours’ TWA for workers who assisted with cleaning was 11 ppm (range = 0.5~59 ppm) in 1994 [14]. In 1994, the GM of the 8 hours’ time weighted exposure level of workers using TCE was 9.9~35.3 ppm; 14.2% of workers had exposure levels above the occupational exposure limit set by the government [15]. The exposure level of workers who engaged in semi-automated cleaning was <1 ppm in 1995, but the peak exposure level of TCE (100 ppm) occurred during the replacement of the TCE solution, which was performed once per week. However, the ambient exposure level of TCE for manual cleaning workers was 107 ppm [16].

### Scientific evidences for the causal relationship between trichloroethylene exposure and kidney cancer

#### Meta-analysis

The first meta-analysis was reported in 2011 and included 24 cohort and case–control studies; in the TCE exposed group, the relative risk (RR) was 1.27 and the 95% confidence interval (CI) was 1.13~1.43. The RR of higher exposure group was 1.58 (95% CI = 1.28~1.96) and the strength of association increased in the higher exposure group compared to the lower exposure group [17]. The second meta-analysis, which included 15 cohort and 13 case–control studies, was conducted during 1950~2011 and was published in 2012. In the second analysis, the RR for kidney cancer from the cohort studies, case-controlled studies, and the pooled RR of all studies was 1.26 (95% CI = 1.02~1.56), 1.35 (95% CI = 1.17~1.57), and 1.32 (95% CI = 1.17~1.50), respectively. The authors of this study concluded that significant and strong associations were consistent among the studies to measure exposure levels and also emphasized the possibility of underestimating risk due to the misclassification of exposure (i.e., exposure to TCE was usually broadly measured with exposure to chlorinated hydrocarbon or other organic solvents) [18]. This was a reasonable conclusion considering that differences in carcinogenicity among halogenated hydrocarbons; unsaturated short-chain halogenated hydrocarbons were identified to have carcinogenic effects in animal studies. TCE, an unsaturated short-chain hydrocarbon, has carcinogenic effects, but trichloroethane, which is similar to TCE in chemical structure, is saturated and is not carcinogenic [19].

#### Cohort studies

Most studies regarding the association between TCE exposure and kidney cancer were conducted in aerospace workers in the United States and Demark by measuring their exposure level using a job-exposure matrix (JEM) based on the job name or code. Zhao et al. reported a significant RR (4.90, 95% CI = 1.23~19.60) of incidence in only the high exposure group, which consisted of 5,049 male aerospace workers who work for >2 years during 1950~1993 and were followed during 1988~2000 [20]. In manual workers who worked >5 years and used TCE in Denmark, the RR was 1.5 (95% CI = 1.1~2.2) [21]. Although the RRs in the early cohort study [22] and other subgroups in the same cohorts were statistically insignificant, the dose–response relationship between the risk for kidney cancer and TCE exposure was also identified (Table 6) [20-23].

**Table 6:** Summary of risk measurement of the major cohort and case–control studies

**Authors, (years) country**	**Study subjects/design**	**Exposure measurement**	**Overall OR or RR**	**ORs or RRs according to exposure level**
Moore et al. (2010) [24] Czech Republic, Poland, Romania, Russia	Hospitals in 4 European countries (n = 1,097), 1999–2003; hospital controls with diagnoses unrelated to smoking or genitourinary disorders (n = 1,476)/case–control	Specialized job-specific questionnaire for specific jobs or industries of interest focused on TCE with exposure assignment by frequency and confidence of TCE exposure	1.63 (1.04–2.54) for all subjects 2.05 (1.13–3.73) for high-confidence assessments only	Duration
				<13.5 yrs: 1.89 (0.84–4.28)
				≥13.5 yrs: 2.25 (0.95–5.29)
				<1080 hrs: 1.22 (0.48–3.12)
				≥1080 hrs: 2.86 (1.31–6.23)
				Cumulative
				<1.58 ppm·yr: 1.77 (0.64–4.80)
				≥1.58 ppm·yr: 2.23 (1.07–4.64)
				Average intensity
				<0.076 ppm: 1.73 (0.75–4.02)
				≥0.076 ppm: 2.41 (1.05–5.56)
				*reference group: non-exposed
Chabotel et al. (2006) [25] France	RCC (n = 87), from urologists’ files and area teaching hospitals, 1993–2003; urologist or general practitioner patient controls (n = 316)/case–control	Semi-quantitative cumulative TCE exposure and presence/absence of peak TCE exposure assigned to subjects using a JEM designed using information obtained from questionnaires and routine atmospheric monitoring of workshops or biological monitoring (U-TCA) of workers carried out since the 1960s.	1.64 (0.95–2.84) for full study; 1.68 (0.97–2.91) with 10-yr lag	High cumulative level: 3.34 (1.27–8.74)
				ppm·yrs
				1–154: 0.85 (0.10–7.41)
				155–335: 1.03 (0.29–3.70)
				>335: 3.34 (1.27–8.74)
				peak + cumulative level
				(-)/low-medium: 0.90 (0.27–3.01)
				(+)/low-medium: 1.34 (0.13–14.0)
				(-)/high: 2.74 (0.66–11.4)
				(+)/high: 3.80 (1.27–11.4)
				with 10-yr lag
				high: 2.16 (1.01–4.65)
				+peaks: 3.15 (1.19–8.38)
Zhao et al. (2005) [20] USA	Aerospace workers with >2 yrs of employment at Rockwell/Rocketdyne’s Santa Susana Field Laboratory, 1950–1993, follow up 1950–2001 (mortality, n = 6,044), 1988–2000 (incidence, n = 5,049) /cohort	Using job titles, job codes, dates of employment related with JEM and calculated cumulative intensity scores		mortality medium: 0.85 (0.15–4.93) & 1.69 (0.29–9.70) with 20-yrs lag
				high: 0.96 (0.09–9.91) & 1.82 (0.09–38.6) with 20-yrs lag
				incidence medium: 1.26 (0.26–6.14) & 1.19 (0.22–6.40) with 20-yrs lag
				high: 7.71 (0.65–91.4) & 7.40 (0.47–116)
Brüning et al. (2003) [26] Germany	Histologically confirmed RCC (n = 134), from hospitals, 1992–2000; hospital controls (n = 401)/case–control	Self-reported exposure duration using JEM	2.47 (1.36–4.49)	<10 yr: 3.78 (1.54–9.28)
				10-<20 yr: 3.78 (1.54–9.28)
				≥20 yr: 2.69 (0.84–8.66)
Raaschou-Nielsen et al. (2003) Denmark	Blue-collar workers employed >1,968 at 347 TCE-using companies (n = 40,049; 14,360 with presumably higher-level exposure to TCE). Follow up to 1997/cohort	duration of employment, yrs of 1^st^ employment at a TCE-using company, number of employees in the company	1.20 (0.94–1.50)	≥5 years all subject: 1.6 (1.1–2.2) in
				subcohort with expected higher exposure levels: 1.7 (1.1–2.4)
Pesch et al. (2000) [27] Germany	Histologically confirmed RCC from hospitals (5 regions) (n = 935), 1991–1995; controls randomly selected from residency registries (n = 4,298)/case–control	TCE and other exposures assigned by questionnaire, assessed occupational history using job title (JEM approach)	1.24 (1.03–1.49)	substantial exposure
				men: 1.3 (0.8–2.1)
				women: 1.8 (0.6–5.0)
				high exposure men: 1.1 (0.8–1.5)
				women: 1.8 (0.6–1.9)
				medium exposure
				men: 1.3 (1.0–1.8)
				women: 1.3 (0.7–2.6)
Morgan et al. (1998) [22] USA	Aerospace workers with >6 mths during 1950–1985 at Hughes (Tucson, AZ) (n = 20,503; 4,733 with TCE exposure), follow up 1950–19/cohort	TCE exposure intensity assigned using JEM.	1.14 (0.51–2.58)	High cumulative exposure score: 1.59 (0.68–3.71)

Inconsistencies in the results from the cohort studies could be due to risk underestimation (i.e., non-deferential misclassification of exposure). Most of the authors of the cohort studies measured exposure-related variables using the JEM, which is a type of ecological exposure indicator, and assumed that the exposure level would be consistent for the same job. These inconsistencies could lead to the misclassification of exposure and decreased statistical power in their results.

#### Case–control studies

The main results of the case–control studies are presented in Table 6. According to the case–control studies, consistent associations and dose–response relationships were observed after adjusting for various confounding variables. A review of the classification of exposure levels in individual research papers was important to fully evaluate work-related TCE exposures. According to a recent study by Moor et al., the risk of kidney cancer in the exposure group was twice as high as the non-exposure group. A dose–response relationship was identified in this study; the odds ratio (OR) of the exposed group <13.5 years was 1.89 (95% CI = 0.84~4.28) and ≥13.5 years was 2.25 (95% CI = 0.95~5.29). If the total exposure time was <1080 hours, the OR of the exposed group was 1.22 (95% CI = 0.48~3.12); if the exposure time was ≥1080 hours, the OR was 2.86 (95% CI = 1.31~6.23). An exposure time of 1080 hours is about 27 weeks or 6 months for a 40-hour work-week. A dose–response relationship could be identified for the cumulative exposure level; the ORs were 1.77 (95% CI = 0.64~4.80) and 2.23 (95% CI = 1.07~4.64) for <1.58 ppm・year and ≥1.58 ppm・year, respectively. The ORs of the average exposure level was 1.73 (95% CI = 0.75~4.02) and 2.41 (95% CI = 1.05~5.56) for <0.0076 ppm and ≥0.0076 ppm, respectively, compared with the non-exposure group [24]. Therefore, kidney cancer can possible develop in people with a low cumulative exposure level or a short exposure duration.

In 2006, Charbotel et al. reported the results from case–control studies based on 87 RCC cases and 316 control groups in France. The cumulative exposure levels of TCE were divided into the following 3 groups: low exposure group (1~155 ppm·year), medium exposure group (155~355 ppm·year), and high exposure group (>335 ppm·year). The ORs were determined to be 3.34 (95% CI = 1.27~8.74), 1.03 (95% CI = 0.29~3.70), and 0.85 (95% CI = 0.10~7.41) in the high, medium, and low exposure groups, respectively. Peak exposure was defined as being exposed to 200 ppm of TCE for >15 minutes. The OR for peak exposure was 3.80 (95% CI = 1.27~11.40) [25].

In an earlier case–control study, Brüning et al. evaluated occupational exposure levels of TCE in 134 kidney cancer cases and 401 controls from 1992~2000; the OR of the exposed group was 1.80 (95% CI = 1.01~3.20), and the OR of workers involved with cutting and cleaning was 5.57 (95% CI = 2.33~13.32). The OR of the exposure group <10 years, 10~20 years, and >20 years of exposure was 3.78 (95% CI = 1.54~9.28), 1.80 (95% CI = 0.67~4.79), and 2.69 (95% CI = 0.84~8.66), respectively [26]. Pescht et al. also presented statistically significant increase of OR [27].

In some studies, the possibility of a high-dose phenomenon or genetic sensitivity related to TCE exposure and the risk of kidney cancer has been reported. Therefore, renal tubule toxicity would be more important than genetic toxicity for carcinogenesis. A history of peak exposures was the most important variable related to exposure in another study [28]. The presence of a genetically susceptible population for which the glutathione S-transferase theta 1 enzyme was active has also been reported in a case-controlled study [24].

## Conclusion

The association between TCE exposure and kidney cancer should be definite according to epidemiologic studies. Although the findings from the cohort studies were inconsistent, the results from the case–control studies were consistent. Considering the limitations of cohort studies and the low prevalence of kidney cancer in those studies, the results of the well-designed case–control studies could be more useful for evaluating the causal association between TCE exposure and kidney cancer risk. The study subjects of the cohort studies were restricted to a few jobs or industries (i.e., aerospace workers or workers using TCE in the United Sates and Denmark) and exposure levels were measured based on the JEM, which can possible lead to the misclassification of exposure. According to the results from the meta-analyses and case–control studies, an increased risk for the exposure group and a dose–response relationship was identified using various exposure measures, such as cumulative exposure level, exposure duration, peak exposure, or JEM. Therefore, the epidemiologic evidence for the causal relationship between TCE exposure and kidney cancer could be sufficient.

In Korea, TCE remains a widely used chemical by various manufacturers. Occupational physicians should remember that past exposure levels of TCE could have been very high in patients when evaluating work-related causes of kidney cancer. TCE exposure levels have been as high as >100 ppm in some studies; plating, cleaning, or degreasing were common high-risk jobs for TCE exposure. Furthermore, the exposure level for manual jobs could be higher than automated jobs. The peak exposure level of TCE could also be considered an important variable due to the possibility of carcinogenesis, which is related to high dose phenomenon.

In summary, TCE is a widely used chemical and the incidence of kidney cancer has increased with the development of novel diagnostic techniques. Therefore, the construction of a JEM, case–control studies to evaluate risk factors associated with TCE exposure, and proper preventive policies are necessary. This review could be a comprehensive reference for evaluating the relationship between work-related TCE exposure and kidney cancer in Korea.

## Competing interests

The authors declared that they have no competing interests.

## Authors’ contributions

IK: The first author of this article. She designed this research, collected and interpreted the data, prepared the draft of this manuscript, and approved the final version of the manuscript. JH: He reviewed and interpreted the epidemiologic articles and revised the draft of this manuscript, and approved the final version of the manuscript. JHL: He collected and reviewed the exposure status related report or papers and prepared the draft of the manuscript. KY: He collected and analyzed the exposure measurement data, revised the draft of this manuscript, and approved the final version of the manuscript. JR: The corresponding author of this article. He suggested the design of this research, interpreted data, revised the draft of this manuscript, and approved the final version of the manuscript.
